# The human gut microbiome and its metabolic pathway dynamics before and during HIV antiretroviral therapy

**DOI:** 10.1128/spectrum.02205-24

**Published:** 2025-06-16

**Authors:** Oumar Dolo, Fousseini Coulibaly, Anou M. Somboro, Shan Sun, Modibo Diarra, Aminata Maiga, Soungou Bore, Djeneba B. Fofana, Anne-Genevieve Marcelin, Brehima Diakite, Yaya Kassogue, Jane L. Holl, Vincent Calvez, Cheick B. Traoré, Robert Murphy, Anthony A. Fodor, Mamoudou Maiga, Almoustapha I. Maiga

**Affiliations:** 1 University Clinical Research Center (UCRC), University of Sciences, Techniques and Technologies of Bamako 225803 , Bamako, Bamako Capital District, Mali; 2 Medical Biology Laboratory of the Point G University Hospital Center 665093 , Bamako, Bamako Capital District, Mali; 3 School of Laboratory Medicine and Medical Science, University of KwaZulu-Natal 161892, Durban, KwaZulu-Natal, South Africa; 4 Department of Bioinformatics, University of North Carolina at Charlotte 14727, Charlotte, North Carolina, USA; 5 Research and Training Center for Molecular Pathologies (CREFPAM), University of Sciences, Techniques and Technologies of Bamako 225803 , Bamako, Bamako Capital District, Mali; 6 Virology Department of the “Pitié-Salpêtrière” Hospital, Paris, France; 7 Department of Neurology, University of Chicago 220134, Chicago, Illinois, USA; 8 Institute for Global Health, Northwestern University 3270, Evanston, Illinois, USA; University of Arkansas for Medical Sciences, Little Rock, Arkansas, USA

**Keywords:** antiretroviral therapy, gut microbiota, HIV, Mali

## Abstract

The human gut microbiome consists of highly complex microbial populations that play important roles in disease pathogenesis. Changes in the gut microbiome are associated with HIV infection. However, it is unclear whether gut microbiome dysbiosis is causally linked to disease or whether it simply reflects disease-induced changes in the host immune and metabolic systems. This study aims to describe and compare intestinal microbial compositions and derived metabolic pathways in people living with HIV (PL-HIV) from prior to antiretroviral therapy (ART) to 6 and 12 months after ART initiation, compared to HIV-negative individuals. Whole-genome microbiome sequencing coupled with bioinformatics analysis was used to characterize participants’ intestinal microbial structures and derived metabolic pathways. Pre- and post-ART gut microbiota characterization of PL-HIV revealed substantial dysbiosis compared to HIV-negative people. An enrichment of pro-inflammatory microorganisms was the hallmark of dysbiosis in the PL-HIV pre-ART, with a decline in *Proteobacteria* at 6 months of ART, continuing until 12 months of ART. Lower proportions of *Bacteroidetes* were noted pre-ART, but they increased slightly at 6 months of ART before decreasing again at 12 months of ART. Additionally, we reported metabolic changes that are particularly important for health and are associated with dysbiosis both before and post-ART. Alteration of the pyruvate fermentation to the isobutanol metabolic pathway persisted in PL-HIV after 12 months of ART, and this mechanism was correlated with a decrease in *Ruminococcus bromii* species. ART initiation appears to lead to changes in several crucial metabolic pathways and may not entirely restore the dysbiosis of the gut microbiota caused by HIV.

**IMPORTANCE:**

Researchers are facing a major challenge in the treatment of HIV infection due to the continuous use of antiretroviral (ARV) molecules. However, regularly monitoring these molecules is necessary because they are not without consequences. They have toxicity and side effects and could also destabilize the intestinal microbiota, which could harm the metabolic pathways essential to good health. This study reveals that ARV treatment only partially restores gut microbiota dysbiosis and alters metabolic pathways due to pathogenic taxa. This provides additional insights into the relationship between antiretroviral therapy and the microbiome, potentially leading to new prevention and treatment strategies such as probiotic/prebiotic or microbiota transplants.

## INTRODUCTION

The human gut microbiota consists of highly complex microbial populations with important roles in the mechanisms of pathogen elimination, energy recovery, and immune regulation (1). Differences in the composition of the gut microbiota affect these functions (2, 3). The gut microbiota is a set of microorganisms that inhabit our intestines, including bacteria, viruses, fungi (including yeasts), and parasites. Age, nutrition, treatment, behavior, heredity, and illnesses like HIV, malaria, and tuberculosis all have an impact on its dynamic makeup (4–10).

HIV infection is typified by immuno-stimulatory microbial product translocation and disruption of the gut immune barrier, both of which can result in persistent systemic inflammation. Many recent reports have indicated that the composition of the gut microbiota plays a significant role in these processes (11). Since the availability and widespread use of antiretroviral therapy (ART), HIV mortality and morbidity have decreased dramatically. ART includes nucleoside reverse transcriptase inhibitors (NRTIs), non-nucleoside reverse transcriptase inhibitors (NNRTIs), protease inhibitors (PIs), integrase strand transfer inhibitors (INSTIs), chemokine receptor antagonists, and fusion inhibitors (12). Each ART class targets an important step in HIV replication, from viral attachment through entry to maturation.

Patients must take ART for life, which may damage microorganisms (dysbiosis) living normally and symbiotically in the gut. Yet, HIV mortality remains higher in Africa than in other parts of the world, even among patients on ART (13). Gut dysbiosis may persist even in patients with controlled HIV viral load by ART, who may experience immune suppression, disease progression, and inflammatory unbalance (14).

Most of the work examining the gut microbiota consists of descriptive cross-sectional studies and focuses on bacterial communities only. A study described the evolution of the gut microbiota over time, during ART in people living with HIV (PL-HIV), by only using 16S ribosomal RNA sequencing to analyze bacterial compositions. Another study also investigated the bacteria in the gut and saliva in patients on long-term ART; however, it did not examine any other microbial populations, such as parasites, viruses, and fungi, nor did it provide any functional microbial-metabolic data. Nonetheless, it was reported that ART, particularly NRTI-based ART, has remarkable effects on the fecal bacterial community with a decrease in α-diversity, enrichment of *Prevotella*, and a decrease in Bacteroides over time (15). Furthermore, PL-HIV with viremia suppressed on ART have a microbial composition that is distinct from that of viremic patients and, overall, is more similar to healthy non-HIV-infected individuals despite chronic gut inflammation (16). In a study using rhesus macaques as an animal model, short-term antiretroviral therapy was associated with a brief rise in certain bacterial microbiomes that had been disturbed by simian immunodeficiency virus infection and a better gut microbiota with a composition similar to normal that prevented immunological activation (17). It has been suggested that dysbiosis in long-term ART impacts the essential functions of the gut microbiota (e.g., immunity), which could explain the persistent chronic inflammation often observed in PL-HIV. Antiretroviral therapy plays a vital role in managing HIV, significantly enhancing the health and life expectancy of people living with the virus. Since the adoption of the World Health Organization’s test-and-treat strategy, the number of individuals receiving ART has steadily increased. However, ART is not without side effects; some regimens, particularly those including PIs, can cause adverse gastrointestinal symptoms such as nausea, vomiting, diarrhea, abdominal pain, and even intestinal disorders (18). This study aimed to investigate the composition of the gut microbiome and its associated metabolic pathways in people living with HIV at three key time points: before the initiation of ART, 6 months, and 12 months of treatment. These findings were compared with those from HIV-negative healthy controls. The research was conducted in Mali, a low-income country in Sub-Saharan Africa, a region that remains heavily impacted by the HIV epidemic, accounting for 49% of new infections and nearly 62% of global HIV/AIDS-related deaths as of 2023 (19).

## MATERIALS AND METHODS

### Study design, settings, and population

We conducted a longitudinal cohort study from August 2017 to February 2019 that included two groups of participants (HIV-positive individuals and healthy participants).

**Fig 1:**
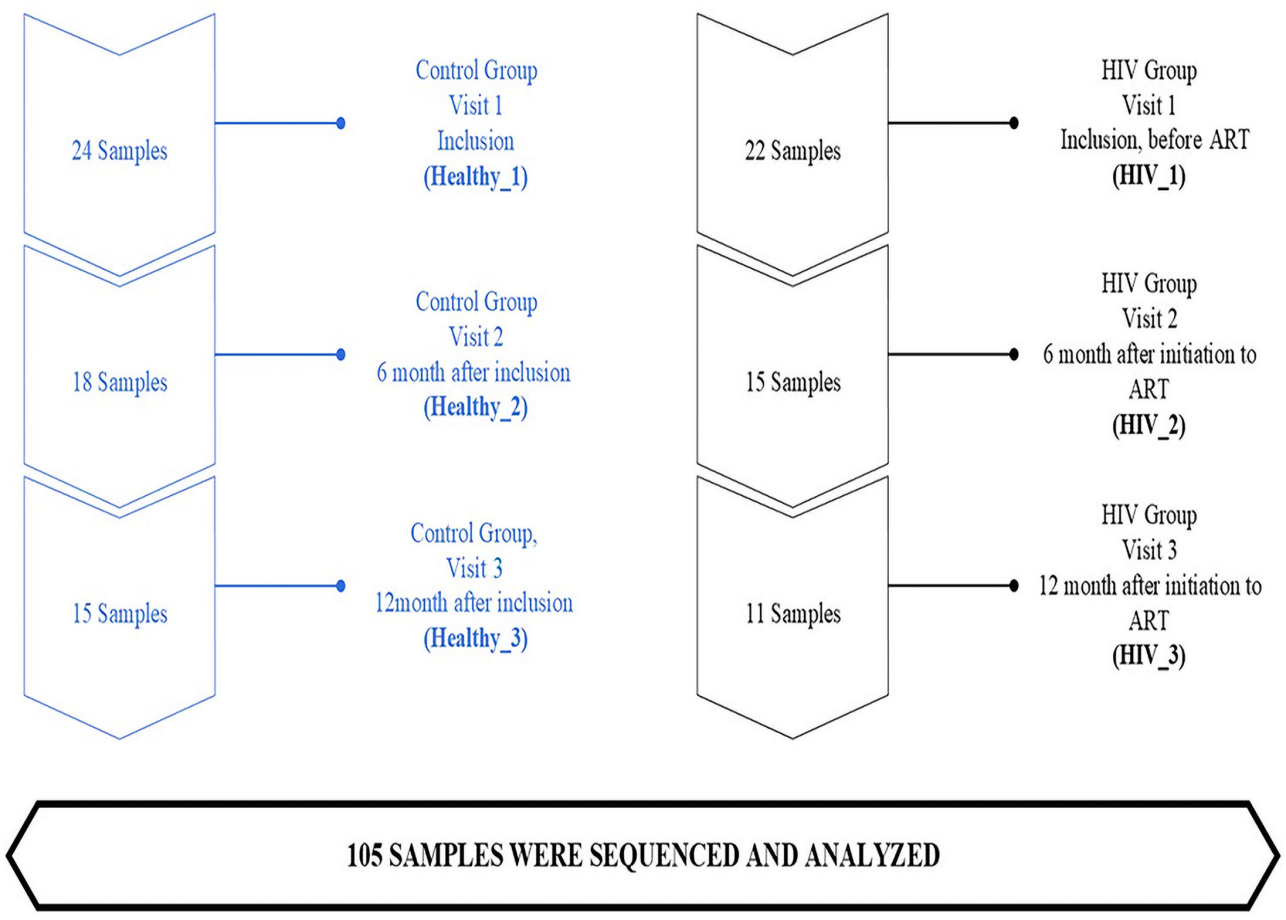
Study populations (sample size and follow-up duration).

The HIV group consisted of individuals newly tested positive for HIV, with a CD4 count below 200 cells/mm³ and antiretroviral therapy-naive at enrollment. People living with HIV (heretofore *“*HIV”) were enrolled at the two HIV Clinical Care Centers in Bamako, Mali, West Africa, namely, the “Centre de Soins, d'Animation et de Conseil pour les personnes vivant avec le VIH/SIDA” and the *“*Centre National d'Appui à la Lutte Contre la Maladie”. Participants were followed from inclusion (i.e*.,* before treatment initiation, which is Month 0), 6 months (M6), and 12 months (M12) after ART initiation. All the HIV-infected individuals received a first-line ART regimen comprising tenofovir (TDF), lamivudine (3TC), and efavirenz (EFV), in line with Mali’s national treatment guidelines, which align with World Health Organization (WHO) recommendations. The healthy group was individuals not living with HIV (heretofore “healthy”) who were enrolled at the Teaching Hospital of Point G in Bamako, Mali. The healthy participants’ group was considered as a control group that included HIV-negative individuals and was also followed at the same time points as the HIV-positive group (i.e., at M0, M6, and M12). The study population, including sample size and follow-up duration, is presented in Fig. 1.

### Inclusion and exclusion criteria

The study included ART-naive HIV-positive individuals with a CD4 count of less than 200 cells/mm³, as well as healthy individuals aged from 18 to 50 years, who were willing and able to provide the required biological samples, undergo HIV testing, and consent to have their samples stored for future research.

Individuals who had initiated ART or antibiotic treatment in the 4 weeks prior to enrollment, those with any acute or chronic infection, or any known medical condition or circumstance that, in the opinion of the recruiting physician, could compromise the individual’s safety or study participation were excluded. HIV-2-infected individuals were also excluded.

### Clinical data and sample collection

An IRB-approved survey form was used during the participant interviews with a clinical investigator to gather information about the participants’ location, age, gender, and occupation, as well as their past medical history, including any history of HIV or ART. Additional information about dietary habits, general lifestyle (e.g*.*, physical activity, alcohol use, and smoking), other medication use, and co-morbidities was gathered because they are known to influence the human gut microbiota. A physical examination was performed to assess weight, height, and BMI. HIV viral loads with testing dates were collected for PL-HIV from the medical record.

### Genomic DNA isolation from stool samples

Fresh stools were collected in sterile containers from each participant and placed on dry ice. Five milliliters of blood was collected from all participants for HIV testing, plasma viral load, and plasma storage for future biomarker studies or inflammatory biomarker assessments. All samples were transported to the University Clinical Research Center laboratory within 2 hours of collection and stored at −20°C prior to any laboratory assessments.

Genomic DNA was extracted from the stored stool samples using the Qiagen QIAamp DNA Stool Mini Kit. Briefly, stool DNA was extracted using the QIAmp DNA Stool Mini Kit (Qiagen, Hilden, Germany) by first weighing the stool (180–220 mg), and the extraction was performed according to the manufacturer’s instructions. The DNA was selectively bound to a silica membrane within a spin column, followed by sequential washes with ethanol-containing buffers to remove contaminants. A Nanodrop One^c^ (Thermofisher Scientific, Madison, WI, USA) was used to measure the extracted DNA in order to assess the DNA concentration and purity prior to sequencing. The purified DNA samples were then sent to the Sequencing Core at the University of Illinois at Chicago for downstream processing (20).

### Whole-genome microbiome sequencing with Illumina HiSeq 4000

Whole-genome metagenomic sequencing was performed using the Illumina HiSeq 4000 platform to analyze the gut microbiota. Library preparation was conducted using the Illumina DNA PCR-Free Library Prep Kit (Cat. No. 20015963), which minimizes amplification bias, thus preserving the original composition of microbial communities. Sequencing was carried out on the Illumina HiSeq 4000 system, a high-throughput platform capable of delivering deep and accurate metagenomic reads. The sequencing workflow includes library normalization, reagent deposition, system calibration, and flowcell loading, enabling comprehensive profiling of the microbiome structure and function.

### HIV viral load testing

Quantification of plasma HIV-1 RNA was performed using the Abbott m2000 system, composed of two fully automated modules: the m2000sp (sample preparation) and the m2000rt (real-time PCR). Following the manufacturer’s standard operating procedures, this system facilitated the isolation, reverse transcription, amplification, and quantification of viral RNA with a lower detection limit of 40 copies/mL. Internal quality controls were incorporated at each step to ensure the precision, reliability, and reproducibility of the results across all tested specimens (21).

### Bioinformatics analysis

Following the shotgun metagenomics approach and whole-genome sequencing, we proceeded to the analysis phase. Initial quality control was performed using KneadData, which removes human DNA and low-quality reads from the sequencing data, an essential step in microbiome studies where host DNA can be predominant (22). For taxonomic profiling, we employed both MetaPhlAn2 and Kraken2. MetaPhlAn2 identifies species using a curated set of clade-specific marker genes derived from approximately 17,000 reference genomes, enabling species- and even strain-level resolution through its StrainPhlAn module. Although based on different algorithms, k-mers for Kraken2 and marker genes for MetaPhlAn2 are both tools that are widely used and often complementary (23). In contrast, Kraken2 uses exact k-mer matching to classify reads by assigning them to the lowest common ancestor of all genomes containing those k-mers, offering rapid and high-accuracy classification (24). For functional profiling, we utilized HUMAnN 2.0, a pipeline that characterizes microbial metabolic pathways by determining their presence and abundance within the community. This analysis provides insights into the functional potential of the microbiome, addressing the critical question: *“What are the microbes doing or capable of doing?”* (25).

### Statistical analysis

**Fig 2:**
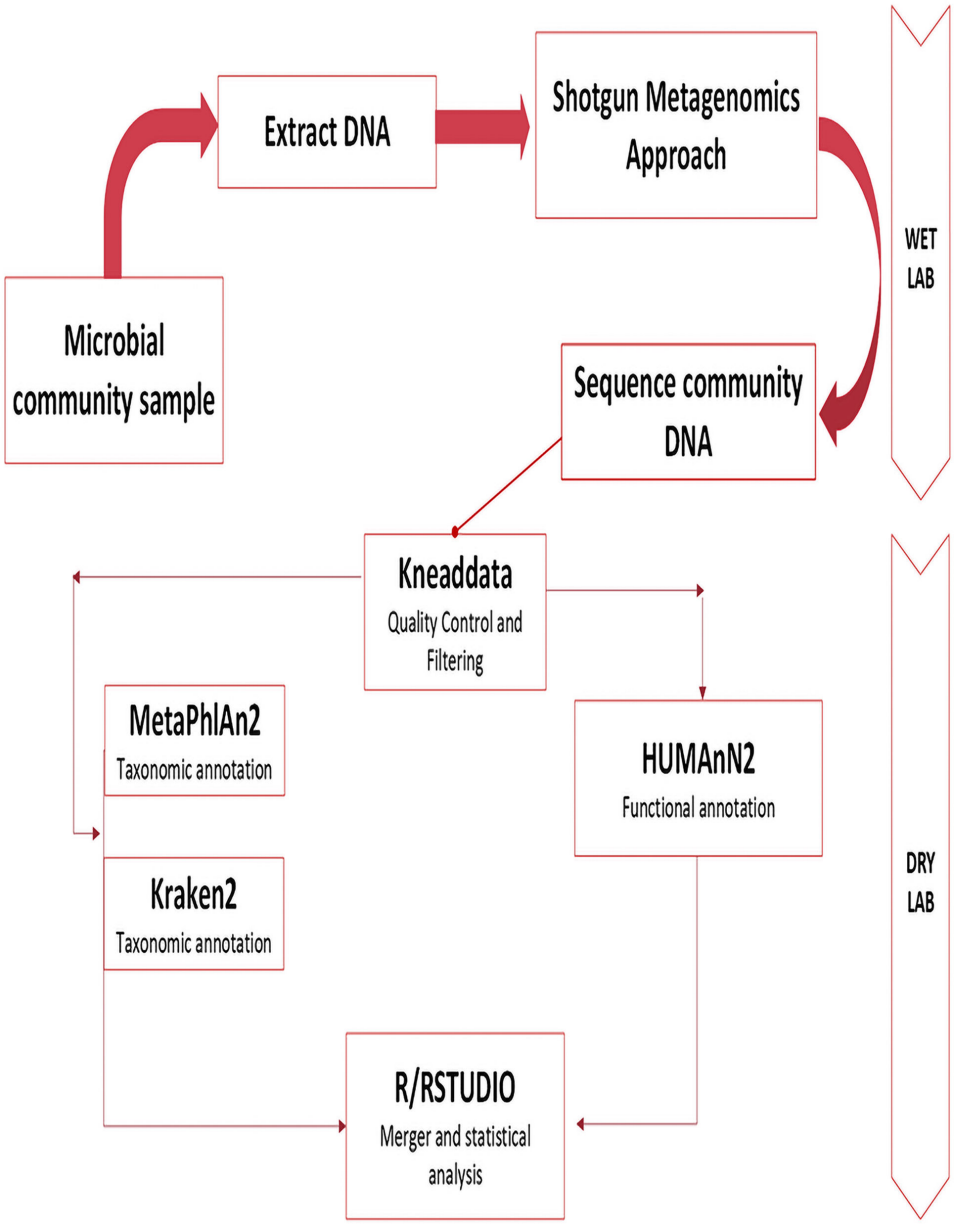
Study workflow.

R/Rstudio software was used to process and analyze our data. Principal coordinate analysis (PCoA) analysis was performed using the function “capscale” in the R package “vegan.” PERMANOVA test was performed using the function “adonis” in the same package. The differential taxa and pathways between HIV and healthy groups were analyzed with the Wilcoxon test. *P*-values were adjusted with the Benjamini-Hochberg method for multiple hypothesis testing. The significance was determined as FDR < 0.1. The taxonomic tree was visualized using the R package “plotmicrobiome.” The study workflow is shown in Fig. 2.

## RESULTS

This study included 46 participants, of whom 22 were HIV positive, and 24 were healthy individuals, serving as controls. Of the 22 HIV participants at the enrollment visit (M0) (HIV_1), 15/22 (68%) were evaluated after 6 months (M6) (second study visit) of ART (HIV_2) and 11/22 (50%) at 12 months (M12) (third study visit) of ART (HIV_3). Healthy participants were followed in a similar manner, at enrollment (Health_1), at 6 months (second study visit, Health_2), and at 12 months (third study visit, Health_3). Of the 24 healthy participants, 18/24 (75%) were evaluated at 6 months (Healthy_2) and 15/24 (62.5%) at 12 months (Healthy_3) (Fig. 1). Stool and blood samples were collected from participants at each study visit to characterize the composition and evolution of the gut microbiome at each follow-up time point and monitor the health status of each participant.

### Sociodemographic and clinical characterizations of the study participants

The mean age of HIV participants was 36.58 (±10.34) years and 25.81 (±5.73) years for healthy participants. Females represented 69.18% of the HIV group with a male/female ratio of 0.33 and 33.33% of the healthy control group with a male/female ratio of 1.89. The mean body weight for the HIV group was 61.57 (± 12.85) kg and 67.69 (± 11.48) kg for the control group, as shown in Table 1. All participants (100%) in both groups reported having a cereal-based diet and residing in Bamako, the capital city of Mali. All HIV participants were self-reported non-smokers, while 23% of healthy participants were self-reported smokers. HIV participants were married (59.9%), single (18.18%), and widowed or divorced (22.72%), while 41.66% of healthy participants were married, 54.16% were single, and 4.16% were widowed or divorced (Table 1). All HIV participants received counseling and were prepared to start the ART triple regimen (tenofovir + lamivudine + dolutegravir [TDF + 3TC + DTG]), as recommended by the National AIDS Protocol of Mali, based on WHO recommendations.

**TABLE 1:** Sociodemographic and clinical parameters of study participants

ID		Cohort 1 (HIV group)	Cohort 2 (healthy control group)
**Participants of each group (Inclusion)**		22	24
**Sex (Female)**		15 (68.18%)	8 (33.33%)
**Mean of age**		36.58 (±10.34) years	25.81 (±5.73) years
**Mean of weight**		61.57 (±12.85) kg	67.69 (±11.48) kg
**Diet**		All cereal based	All cereal based
**Smoking**		0.00%	25.00%
**Residence**		Bamako	Bamako
**Therapy ART**		TDF + 3TC + DTG	N/A
**Marital status**	Married	59.09%	41.66%
	Single	18.18%	54.16%
	Widow/divorced	22.72%	4.16%

**Fig 3:**
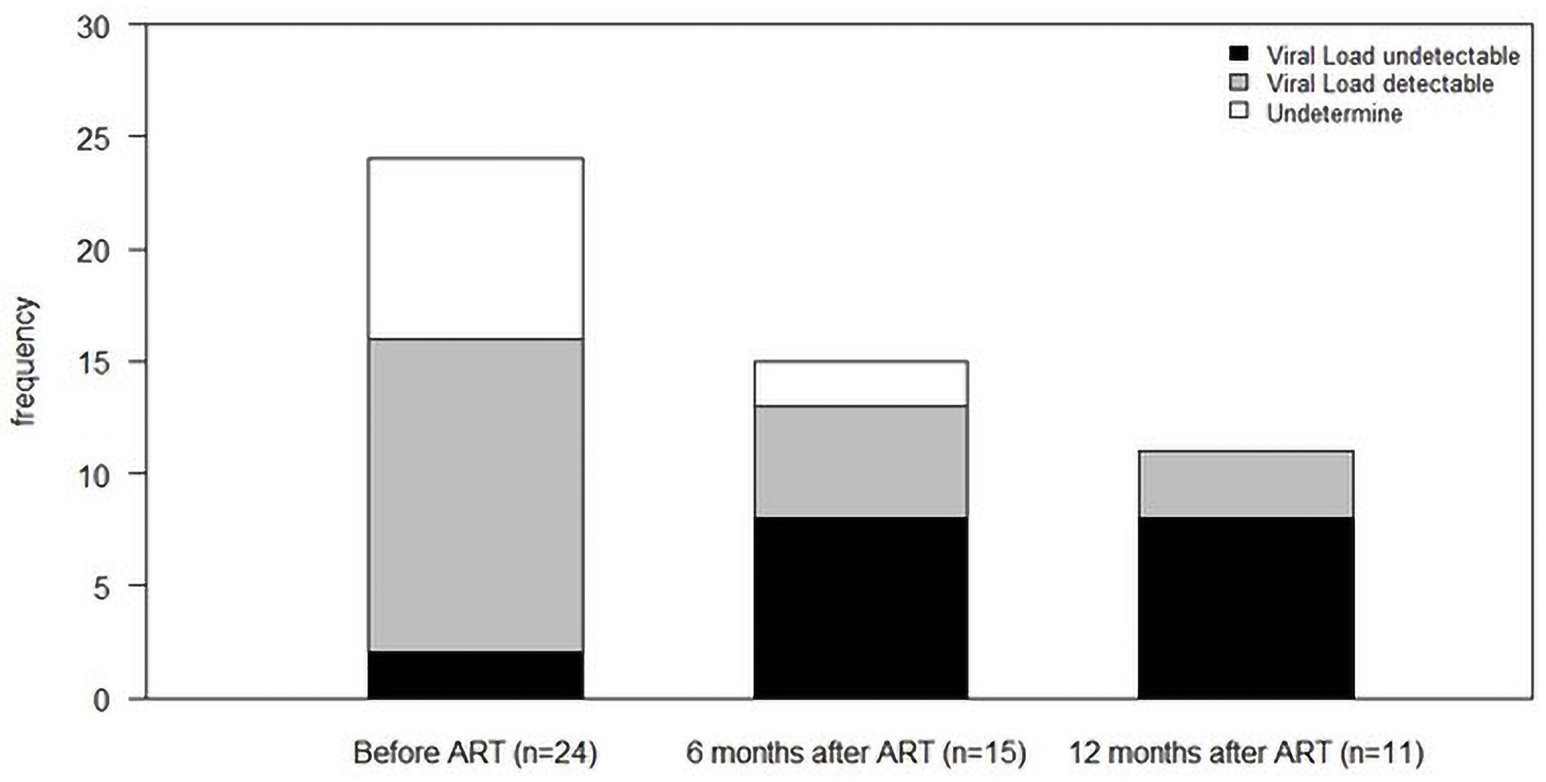
Distribution of participants in the HIV group according to viral loads per visit.

The trend in HIV viral load (before ART, after 6 and 12 months of ART) is shown in Fig. 3. At enrollment, 2 HIV participants had undetectable viral loads (≤50 copies/mL), 14 had detectable viral loads (>50 copies/mL), and 8 were undetermined. After 6 months of ART, there were eight undetectable viral loads, five detectable, and two undetermined, while after 12 months of ART, eight had undetectable viral loads and three were detectable (Fig. 3).

### Composition and diversity of the gut microbiota

**Fig 4:**
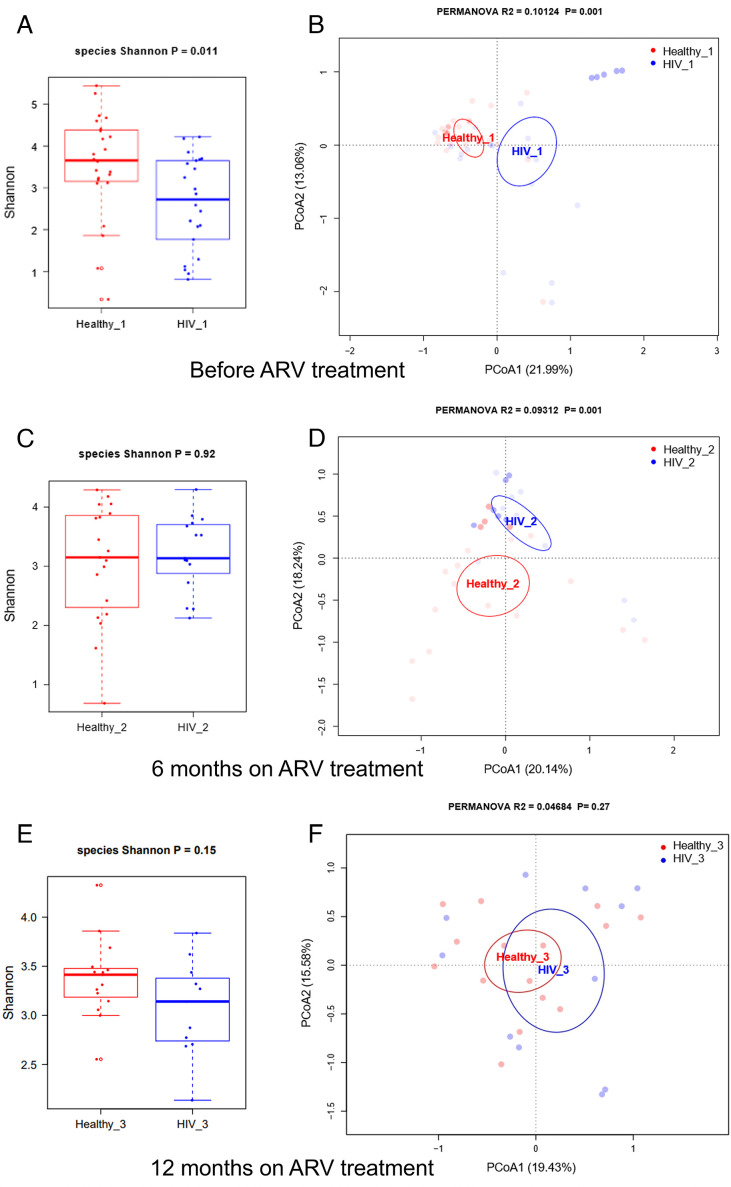
Differences in alpha diversity index and beta diversity at the species level between healthy controls and HIV-infected patients before treatment and after 6 (M6) and 12 months (M12) of treatment. (A) Shannon diversity index and (B) PCoA before treatment. (C) Shannon diversity index and (D) PCoA at 6 months after treatment. (E) Shannon diversity index and (F) PCoA at 12 months after treatment. *R*^2^ and *P*-values in PCoA plots were calculated using the PERMANOVA test. Kraken2 was used for taxonomic annotation. The ellipse of each sample’s 95% confidence interval was used to depict sample clustering in the results, which were plotted based on the first two principal coordinates.

To understand the impact of ART on the gut microbiota and whether ART was associated with restoration of the microbiome, we compared alpha and beta diversity at the species level between HIV and healthy control groups before ART, as well as 6 and 12 months after ART. Before the initiation of ART, the HIV group showed statistically lower α-diversity than the control group (*P* = 0.011) (Fig. 4A). After 6 months of ART, the α-diversity was not significantly different in the HIV group compared to the Healthy control group (*P* = 0.92) (Fig. 4C). A lower α-diversity in the HIV group was observed at 12 months after the start of ART, but the differences were not statistically significant (*P* = 0.15) (Fig. 4E).

Principal coordinate analysis showed that the two groups, HIV and healthy controls, were distinct before the initiation of ART (PERMANOVA, *P* = 0.001) and at month 6 (PERMANOVA, *P* = 0.001) but not at month 12 (PERMANOVA, *P* = 0.27) (Fig. 4).

### Changes in gut microbiota at different taxonomic levels (phylum, genus, and species)

**Fig 5:**
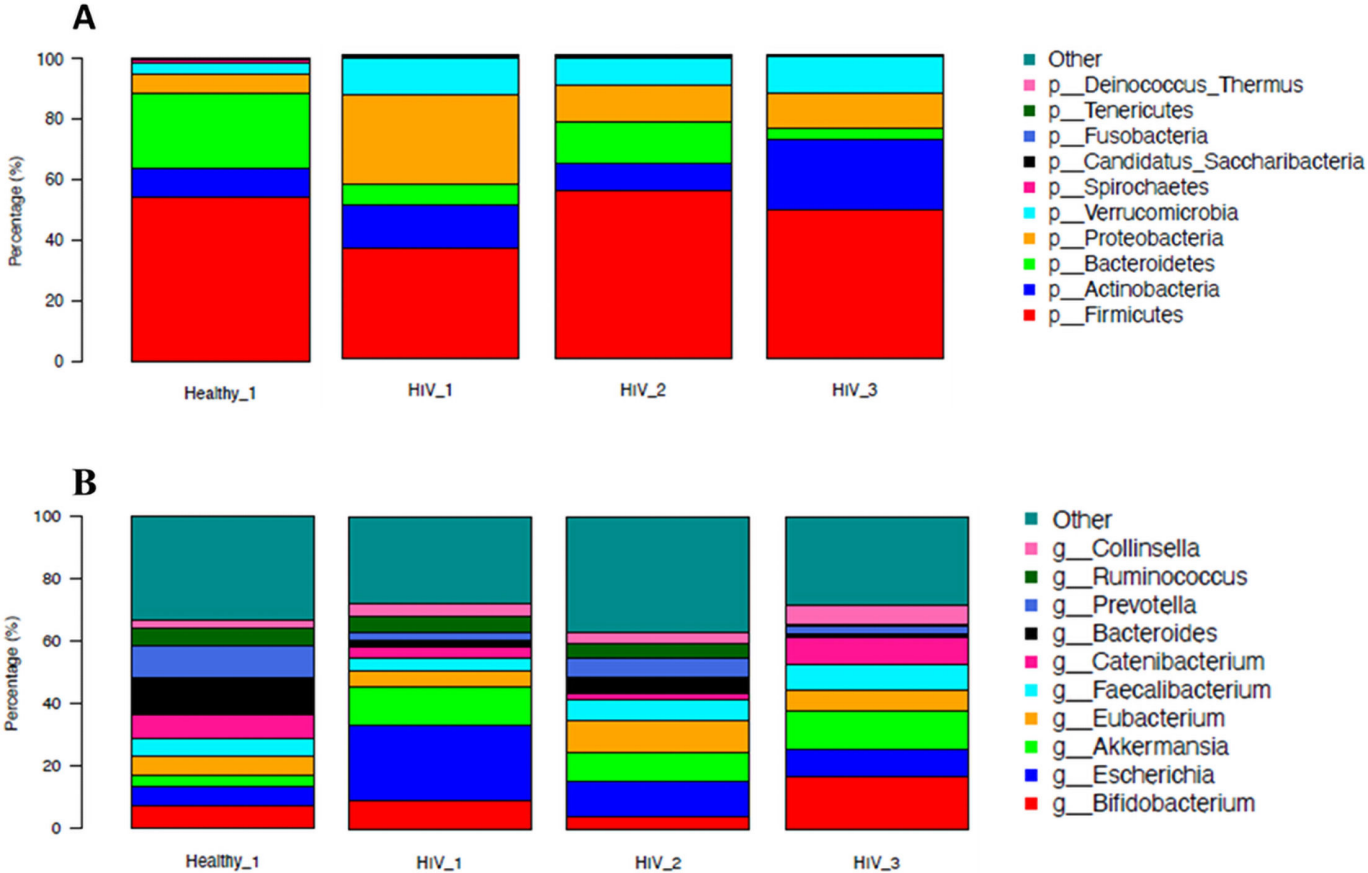
Taxonomic distribution at phylum and genus level of gut microbiota of healthy control group (Health_1) and HIV groups (ART naive [HIV_1], at 6 months on ART [HIV_2], and at 12 months on ART [HIV_3]). (**A**) Mean abundance for each group at the phylum level and (**B**) mean abundance for each group at the genus level. Kraken2 was used for taxonomic annotation.

In order to understand which taxa drove differences between groups, we used Wilcoxon tests to compare HIV groups and healthy controls at each visit and compared the HIV groups at different visits at the phylum and genus levels. The overall gut microbiome was dominated by the phyla *Firmicutes, Bacteroidetes*, *Actinobacteria*, *Proteobacteria,* and *Verrucomicrobia* (Fig. 5A).

We observed that *Bacteroidetes,* which were much more abundant in healthy controls at inclusion than in the HIV group before the initiation of ART (FDR < 0.1), increased in the HIV group after 6 months of ART before decreasing again after 12 months of ART, but this increase was not statistically significant. In contrast, *Proteobacteria* were significantly more abundant in the HIV group before the initiation of ART compared to the healthy control group at inclusion (FDR < 0.1). It decreased in the HIV group after 6 months of ART, and even more in the HIV group after 12 months of ART, but still not as low abundance as in the healthy control groups (Fig. 5A). However, this decrease in abundance was also not significant.

The dominant genera are *Bacteroides*, *Faecalibacterium, Bifidobacterium*, *Prevotella*, *Akkermansia*, *Escherichia,* and *Clostridium* (Fig. 5B). Compared to the healthy control group at inclusion, *Escherichia* was more abundant in the HIV group before initiation of ART, while *Bacteroides*, *Clostridium,* and *Prevotella* were less abundant (FDR < 0.1). The abundance of *Escherichia* decreased from the HIV group before initiation of ART to the HIV group after 12 months of ART, but it was not statistically significant. Future studies with more participants may be needed to increase the statistical power for analyzing the abundance change of *Escherichia*.

**Fig 6:**
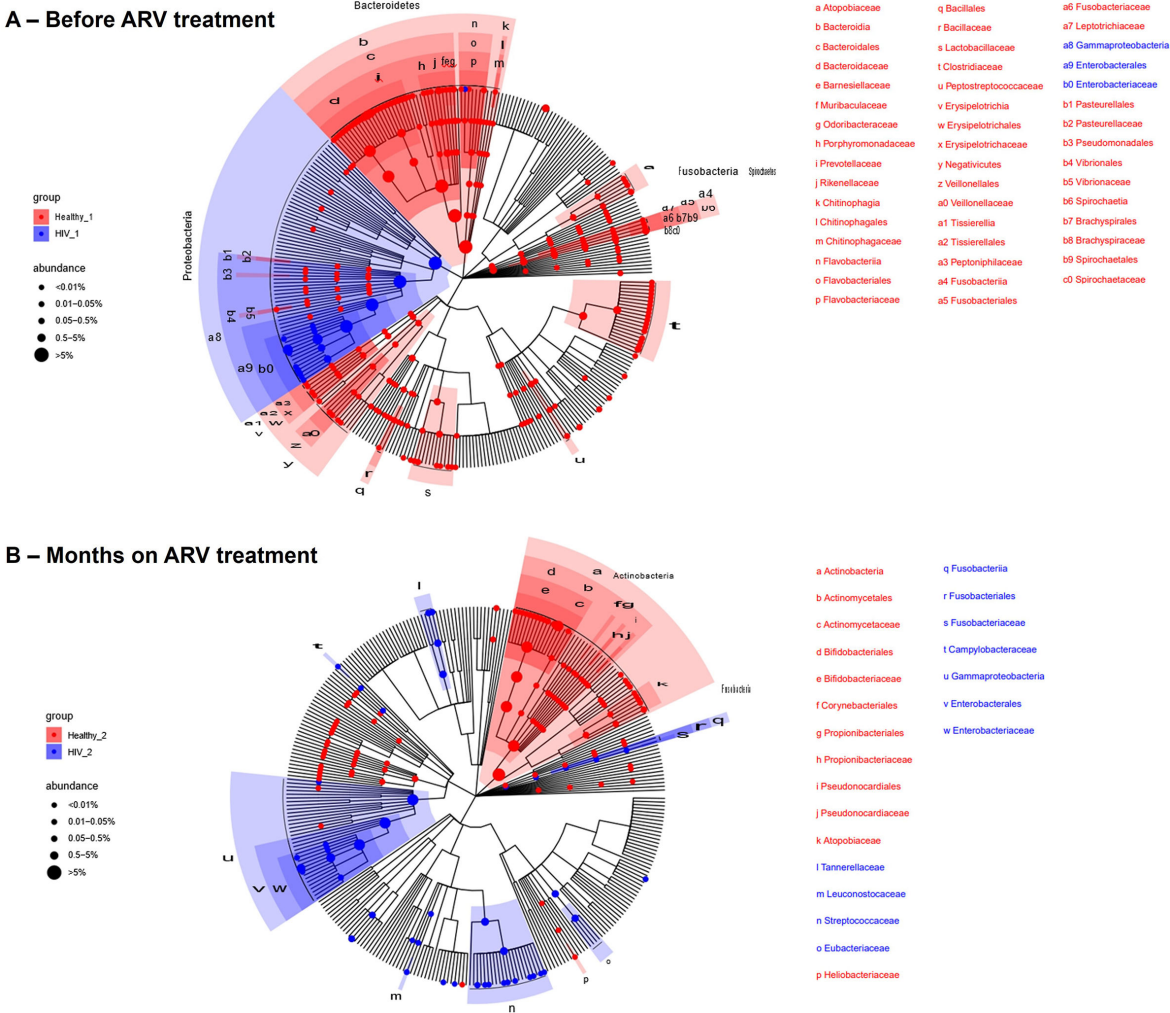
Taxonomic differences between fecal microbiota of HIV groups compared to healthy controls by visit. (**A**) Between the ART-naive HIV group and the healthy control group (HIV_1 and Healthy_1), (**B**) between the HIV groups at 6 months of ART and the healthy control group (HIV_2 and Healthy_2). Wilcoxon test was performed using Kraken2 for taxonomic annotation. *P*-values were adjusted with the Benjamini-Hochberg method for multiple hypotheses testing. Taxa with an FDR < 0.1 are shown in the figure.

Differentially abundant microbial communities were identified in the healthy controls at inclusion and in the HIV groups before initiation of ART (FDR < 0.1). Several members of *Bacteroidetes, Clostridiales*, and *Ruminococcaceae* were more abundant in the healthy control group at inclusion, which are known to be beneficial or protective factors that are generally associated with a great clinical outcome. However, *Gammaproteobacteria* (*P* < 0.05), *Enterobacterales* (*P* < 0.05), and *Enterobacteriaceae* (*P* < 0.05) were more abundant in the HIV group before initiation of ART than in the healthy control group at inclusion (Fig. 6A). We observed a slight restoration of the composition of the intestinal microbiota in the HIV group after 6 months of ART. In addition to *Gammaproteobacteria, Enterobacterales,* and *Enterobacteriaceae*, we observed abundant microbial communities of *Streptococcaceae* (*P* < 0.05)*, Fusobacteriaceae* (*P* < 0.05), and *Campylobacteraceae* (*P* < 0.05) (Fig. 6B). A trend toward normality was seen in the HIV group after 12 months of ART, but the difference between the HIV and the healthy control groups after 12 months of inclusion was statistically insignificant.

### Alterations in functional gut microbiota pathways in the HIV groups

**Fig 7:**
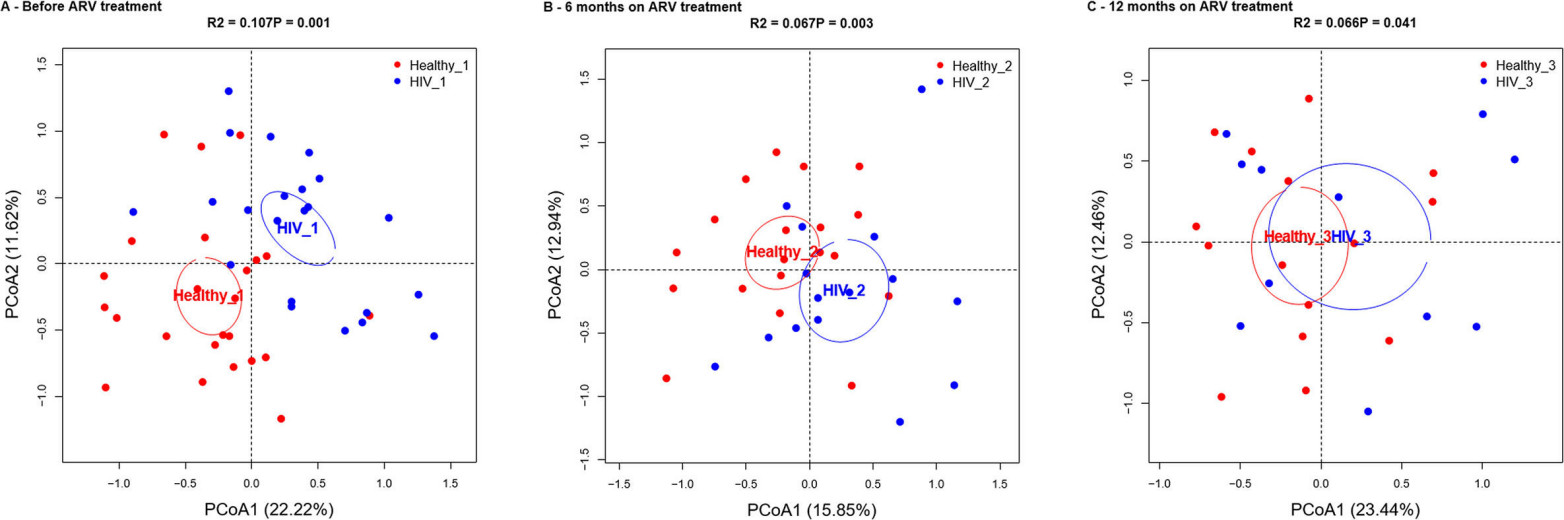
Principal coordinate analysis of functional pathways of all samples by visits, with *R*^2^ and *P*-value from the PERMANOVA test. (**A**) Before treatment (HIV_1 and Healthy_1), (**B**) at 6 months on ART (HIV_2 and Healthy_2), and (**C**) at 12 months on ART (HIV_3 and Healthy_3). HUMAnN2 was used for functional annotation.

**Fig 8:**
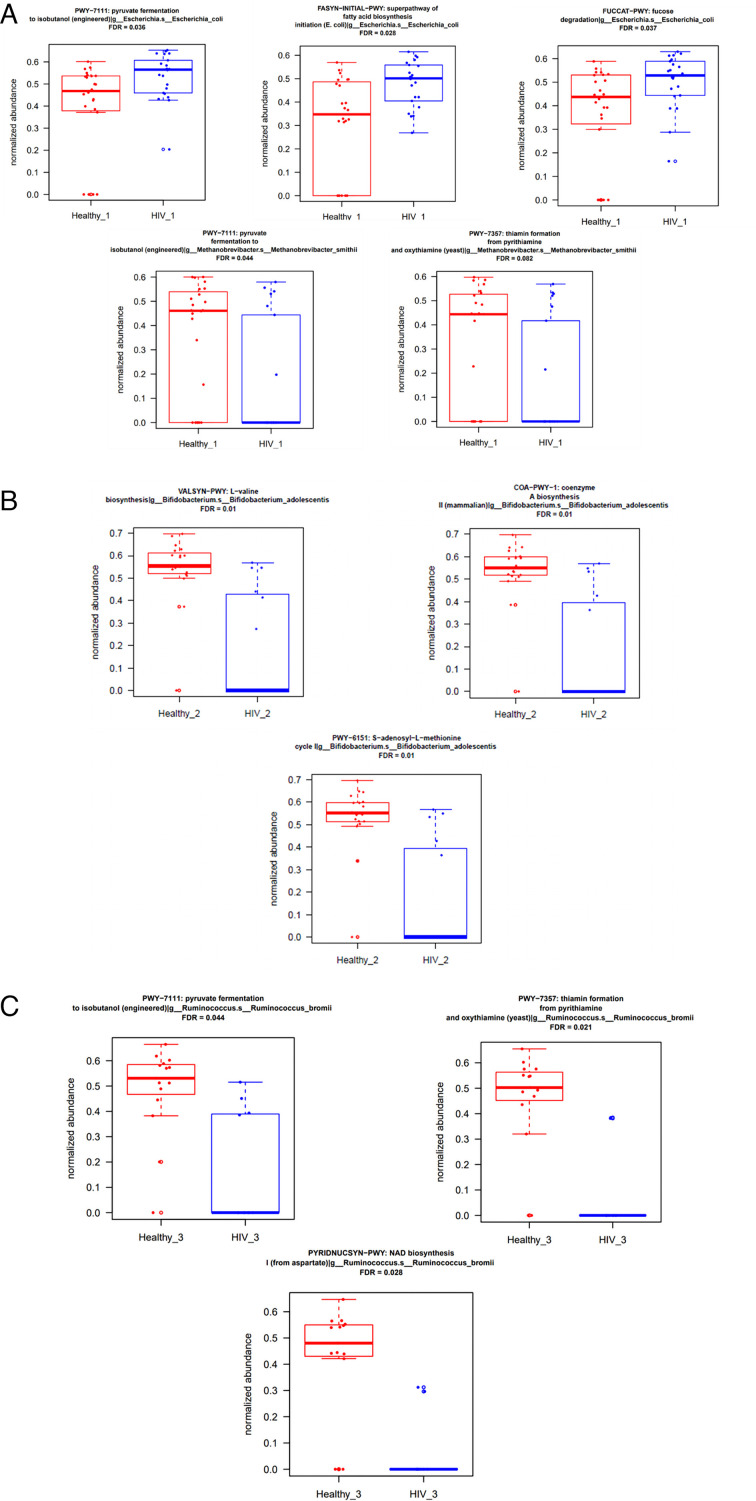
Alteration of functional pathways stratified by microbial species between the two populations studied according to the visits. (**A**) Before treatment (HIV_1 and Healthy_1), (**B**) at 6 months on ART (HIV_2 and Healthy_2), and (**C**) at 12 months on ART (HIV_3 and Healthy_3). HUMAnN2 was used for functional annotation.

In addition to taxonomic classification, we also assigned reads to functional groups using the HUMAnN package. Similar to the results we observed at the taxonomy level, the differences between groups became less pronounced with more time following treatment (Fig. 7). To understand the contribution of individual metabolic pathways to these differences, we again used Wilcoxon tests to compare HIV and healthy control groups at each visit. The abundance of PWY-7111 (pyruvate fermentation to isobutanol) with FDR (0.036), FASYN-INITIAL-PWY (superpathway of fatty acid biosynthesis initiation) with FDR (0.028), and FUCCAT-PWY (fucose degradation) with FDR (0.037) in *Escherichia coli* species was higher in the HIV group before initiation of ART compared to the healthy group at inclusion. The abundance of PWY-7111 (pyruvate fermentation to isobutanol) with FDR (0.044) and PWY-7357 (thiamin formation from pyrithiamine and oxythiamine) with FDR (0.082) in *Methanobrevibacter smithii* species was lower in the HIV group before initiation of ART (Fig. 8A).

At the 6 month follow-up visit after initiation of ART, the abundance of VALSYN-PWY (L-valine biosynthesis), COA-PWY-1 (Coenzyme A biosynthesis II), and PWY-6151 (S-adenosyl-L-methionine Cycle I) with an FDR of 0.1 each in *Bifidobacterium_adolescentis* was lower in the HIV group compared to the healthy control group 6 months after inclusion (Fig. 8B).

At the 12 month follow-up visit, the abundance of PWY-711 (pyruvate fermentation to isobutanol) with FDR (0.044), PWY-7357 (thiamin formation from pyrithiamine and oxythiamine) with FDR (0.021), and PYRIDNUCSYN-PWY (NAD biosynthesis I) with FDR (0.028) in *Ruminococcus_bromii* species was lower in the HIV group compared to the healthy control group 12 months after inclusion (Fig. 8C).

## DISCUSSION

There is mounting evidence that the gut microbiota plays a major role in regulating human health (26–28). The gastrointestinal tract is one of the primary immunological areas and the source of CD4+ T cells. The gut microbiota has a profound effect on immune response pathways and cell networks (29), and HIV infection is associated with altered gut microbiota composition. Nevertheless, there are still unanswered questions regarding the rate at which microbial dysbiosis develops and the potential for antiretroviral therapy to modify the gut microbial communities further. From a comparative analysis perspective, both between and within groups and time points, we observed a substantial decrease in α-diversity indices in HIV individuals, both before and after ART, suggesting that ART may have an impact on intestinal richness and diversity. Our findings are aligned with other recent studies that also found a significant decrease in α-diversity in HIV individuals both before and after ART, when compared to healthy individuals. These studies also suggested that the effects of ART are more closely associated with NNRTIs and PIs than with NRTIs and INSTIs (17, 30, 31). Numerous research studies have investigated the makeup of the gut microbiota in HIV individuals and how it might contribute to the infection’s pathophysiology. However, most of these studies were cross-sectional, focusing on people with advanced HIV infection (32, 33). We found that the gut microbiota composition of the HIV groups (before initiation of ART, 6 months of ART, and after 12 months of ART) differs from the composition of the healthy control groups (at inclusion, 6 months after inclusion, and 12 months after inclusion). Our findings demonstrate that the intestinal environment of dysbiosis, created during the initial acute phase of HIV infection, is only partially restored throughout treatment. Prior studies have demonstrated that dysbiosis is partially restored in HIV individuals during ART (17, 30). In our study, the metagenomic analysis revealed the presence of five major phyla (*Firmicutes, Bacteroidetes, Actinobacteria, Proteobacteria*, and *Verrucomicrobia*) among the six described by Qin et al. (34) in 2010. Previously, the abundance of *Firmicutes*, *Bacteroidetes*, and *Actinobacteria* was reported in a study of treated and untreated HIV infection compared to healthy controls as the most common phyla of all microbes in all groups (35). The study also identified an increased abundance of the genera *Faecalibacterium* and *Bifidobacterium* in the HIV group compared to the control group. These findings are consistent with those reported by Narayanan et al. (36), who similarly observed elevated levels of these genera in the gut microbiota of people living with HIV. Their study further suggests that specific microbial markers may be associated with the type of antiretroviral therapy, particularly among individuals receiving integrase strand transfer inhibitors (36). Interestingly, we found no significant differences between the HIV and healthy individuals at different time points in terms of the abundance of the phylum *Firmicutes*. However, we observed a higher abundance of *Bacteroidetes* members and a lower abundance of *Proteobacteria* members in HIV groups at 6 and 12 months of ART compared to the HIV group before initiation of ART, which could be attributed to the fact that the study included some individuals with high viral load (30). However, the small sample size precludes any further comment on other factors that may influence gut microbiota. In the HIV group at 6 months of ART, we observed a slight increase in the relative abundance of the *Bacteroidetes* phylum and a decrease in *Proteobacteria* compared to the HIV group before initiation of ART, which may suggest a partial restoration of the microbiota. We also observed, in the HIV group at 12 months of ART, a decrease in the relative abundance of the *Bacteroidetes* phylum. However*, Proteobacteria* continued to decrease, which could be explained by the bactericidal activity of particular ART, such as efavirenz (30). Similar findings have been reached in other studies that concluded that ART cannot continually rebuild the gut microbiota (30, 37).

At enrollment, we observed differently abundant microbial populations in the HIV and healthy groups. Numerous *Bacteroidetes, Clostridiales*, and *Ruminococcaceae* species, which are advantageous or protective elements and are typically linked to excellent clinical outcomes, were more prevalent in the control group. In contrast, the HIV group at 6 months of ART showed increased levels of *Streptococcaceae*, *Fusobacteriaceae*, and *Campylobacteraceae* in addition to higher concentrations of *Gammaproteobacteria*, *Enterobacterales,* and *Enterobacteriaceae* in the phylum Proteobacteria than the healthy control group at 6 months of inclusion. This composition of the microbiota, characterized by a depletion of *Bacteroidetes, Clostridiales,* and *Ruminococcaceae*, has been described mainly in human populations infected with HIV before ART initiation through several studies (17, 31, 38). Dinh et al. (39) observed an enrichment of *Gammaproteobacteria, Enterobacterales,* and *Enterobacteriaceae* in untreated PL-HIV compared to healthy individuals. It is important to note that all the HIV participants in our study initiated their treatment with TDF + 3TC + EFV and continued with this regimen until the end of this study. *Prevotella*, *Bacteroides*, and *Escherichia coli* isolates were among the bacteria against which zidovudine was shown to be broadly effective. Furthermore, it was demonstrated that EFV inhibits the growth of three different bacterial taxa: *Enterococcus faecalis*, *Prevotella* species, and *Bacteroides* species. Furthermore, compared to other classes, NNRTI treatment is linked to a particular microbial profile (30). This suggests an incomplete restoration of the intestinal microbiota in HIV individuals despite antiretroviral treatment.

The comparative analysis also revealed a relatively high abundance of *Escherichia coli* in the HIV group before initiation of ART, which is one of the pathogens causing AIDS-associated gastrointestinal symptoms (3). The most abundant species in the healthy control group at inclusion were *Prevotella copri* and *Bifidobacterium adolescentis*. These bacteria tend to positively regulate inflammatory pathways.

It has long been recognized that short-chain fatty acids (SCFAs) have anti-inflammatory qualities and serve as a significant energy source for the colonic mucosa’s homeostasis in a healthy human gut (40, 41). The study suggests that the richness of *Escherichia coli* species in the HIV group before initiation of ART may account for the change in the expression of the super pathway of fatty acid biosynthesis initiation (FASYN-INITIAL-PWY). Depending on pH and concentration, SCFAs significantly affect bacterial growth and virulence; low ileal concentrations enhance the growth of *E. coli,* while higher colonic concentrations significantly inhibit their growth and negatively regulate the expression of virulence genes (fliC, ipaH, FimH, and BssS) (42).

The HIV group before initiation of ART presented alterations in the metabolism of pyruvate, one of the genes belonging to the energy process; these alterations were associated with both an increase in *Escherichia coli* and a reduction of *Methanobrevibacter smithii*. Additionally, compared to healthy controls, the degradation of one of the sugars (fucose) frequently used by the intestinal microbiota as a source of carbon and energy (43, 44) was significant in the HIV group before initiation of ART. This could be explained by the findings suggesting that the accumulation of fuculose-1-phosphate causes the conversion of *Escherichia coli* species to ribose in the intestine, which could reinforce the nutritional niche theory and has heavy responsibilities for the stability of commensal flora in the intestine. *E. coli* and maybe other gut microflora members need multiple limiting nutrients at the same time for growth (45–47). The alteration of the FUCCAT-PWY (fucose degradation) metabolic pathway observed in the HIV group before initiation of ART could be explained by the enrichment of *E. coli* species, which is harmful to intestinal health.

The study revealed that the HIV group at 6 months of ART had alterations in several metabolic pathways essential for good health, including the S-adenosyl I-L-methionine cycle, biosynthesis of coenzyme A-II, and L-valine biosynthesis. These alterations could be explained by a decrease in the species *Bifidobacterium adolescentis,* which is generally considered nonpathogenic (48). S-adenosylmethionine is a natural substance found in almost all bodily tissues and fluids. It is involved in several important processes. As an integral part of the immune system, S-adenosylmethionine preserves cell membranes and facilitates the synthesis and breakdown of chemical molecules in the brain, including dopamine, serotonin, and methylation. This could promote the degradation of immune defenses, knowing that HIV selectively attacks TCD4+ lymphocytes to be able to infect its host (48).

Due to their high metabolic activity, *Bifidobacterium adolescentis* may be a model producer of gamma-aminobutyric acid in the human gastrointestinal system. This suggests that they may interact with the gut-brain axis and have antidepressant properties (49). Its reduction could, therefore, open the door to several metabolic disorders, particularly the alteration of the biosynthesis of coenzyme A-II, which is an acyl group transfer coenzyme involved in numerous metabolic pathways, including the Krebs cycle and beta-oxidation.

The alteration of the metabolic pathway of pyruvate fermentation to isobutanol continued, and the HIV group at 12 months of ART correlated with a decrease in the *Ruminococcus bromii* species. This species was also associated with a decrease in other important metabolic pathways, including thiamine formation from pyrithiamine and oxythiamine and NAD I biosynthesis. Nicotinic acid and thiamine have been shown to have anti-inflammatory effects in male albino rats with carrageenan edema, formaldehyde edema, and cotton ball granuloma (50).

One of the most crucial vitamins for preserving the healthy operation of most living beings is thiamine, which plays a significant co-enzymatic and non-enzymatic role in controlling basic metabolism (51). Furthermore, it should be mentioned that enough thiamine intake is crucial for the healthy operation of the neurological, cardiovascular, and locomotor systems due to its role in glucose metabolism and bioenergetic activities (52). According to our findings, the relative abundance of *Ruminococcaceae* is controlled by the amount of vitamin B1 in the diet. It has also been shown that these bacteria require vitamin B1 in the diet since they lack the *de novo* pathway for vitamin B1 production (53).

Using state-of-the-art metagenomics, we carried out an innovative study on the gut microbiota of HIV patients before and during ART. In this longitudinal study, HIV and healthy individuals were recruited, and they were followed up for 12 months after the start of treatment to analyze the dynamics of microbial populations, their roles in pathogenesis during HIV infection, and their impacts on metabolic pathways essential for health. The findings from this study will inform the development of future host microbiota-directed therapies to control overall inflammation during ART treatment and improve treatment efficacy. Our study’s limited sample size restricted our ability to establish robust associations between immunological, virological, and clinical parameters, which would have offered a more comprehensive understanding of gut microbiota dynamics in our population. Additionally, it did not account for important sociodemographic and environmental factors such as diet, smoking, BMI, sex, and age that are known to influence microbiota composition. All participants living with HIV received the same ART regimen (TDF + 3 TC + EFV), preventing any assessment of the specific effects of different ART classes on gut flora. Future studies with larger, more diverse cohorts and a broader range of clinical, demographic, virological, immunological, and therapeutic variables will be essential to better understand how various factors, including ART regimens, affect the gut microbiota in people living with HIV.

### Conclusion

Our comparative analyses showed that gut microbiome dysbiosis can be observed before ART initiation in PL-HIV, and it is only partially repaired after 12 months of ART with two types of ARVs: NNRTIs and NNRTIs (TDF + 3TC + EFV). Comparing the HIV group with the healthy controls, we discovered that the HIV group had higher abundances of two phyla, *Proteobacteria* and *Verrucomicrobia,* which are considered to be hazardous to health. From the initiation of ART to the 12 month mark, there were significant changes in some important metabolic pathways of pathogenic taxa. Future investigation will be necessary to provide insights into the possible impact of other ARV classes on the gut microbiome.

## Data Availability

The data sets generated and/or analyzed in this study are available from the corresponding author upon reasonable request and with the permission of FMPOS/USTTB Ethic Committee. Sequences from this study are available on NCBI and can be accessed with BioProject ID PRJNA1257948 (https://www.ncbi.nlm.nih.gov/bioproject/1257948).
